# Coronavirus Disease-19 Infection: Implications on Male Fertility and Reproduction

**DOI:** 10.3389/fphys.2020.574761

**Published:** 2020-11-17

**Authors:** Annalisa Navarra, Elena Albani, Stefano Castellano, Luisa Arruzzolo, Paolo Emanuele Levi-Setti

**Affiliations:** Humanitas Clinical and Research Center – IRCCS, Rozzano, Milan

**Keywords:** SARS-CoV-2, male fertility and reproduction, ACE2 and TMPRSS2, Covid-19 sexual transmission, SARS-CoV-2 gender susceptibility

## Abstract

The pandemic caused by Severe Acute Respiratory Syndrome coronavirus 2 (SARS-CoV-2) has led to several concerns on male fertility. Nowadays, there are numerous unanswered questions, for example: is the virus present or not in the seminal fluid of infected subjects? Could the seminal fluid represent a way of sexual transmission for the virus? Why do men appear to be more susceptible than women? Several studies have been carried out to ascertain the presence of SARS−CoV−2 in the seminal fluid, with contrasting results; the expression of angiotensin-converting enzyme-2 (ACE2) and transmembrane serine protease 2 (TMPRSS2) in the testes and in the male genital tract led to speculation about the possible presence of the virus in the seminal fluid. However, it was found that ACE2 and TMPRSS2, used by the virus to enter host cells, are expressed differently in certain testicle cells (stem germ cells, Leydig and Sertoli cells), yet the testicle cells in which ACE2 and TMPRSS2 molecules are simultaneously expressed are rare. This fact would suggest that the virus is not able to enter testicular cells, that it is not present in the seminal fluid and that it cannot infect male germ cells. However, the direct influence of SARS-CoV-2 on the testes is still to be evaluated, and recent results are very controversial. SARS-CoV-2 could enter the testicle using alternative paths and lead to alterations in testicular functionality. Another plausible consideration is that the COVID-19 disease could also indirectly cause alterations to testicular activity, since the fever and the cytokinic storm generated by the immune system can lead to damage of the testicular activity, consequently compromising male fertility. Although the literature provides controversial evidence, the purpose of this review is to lend a general overview about the state of the art. Despite the lack of studies, it would represent a starting point for further investigation about the effect of this coronavirus on male fertility.

## Introduction

Multiple cases of severe pneumonia provoked by a β coronavirus termed Severe Acute Respiratory Syndrome coronavirus 2 (SARS-CoV-2) were reported in Wuhan, China, in December 2019 (Wu and McGoogan, 2020). Since then, the virus has spread worldwide to countries including China, Italy, Spain, Iran, and North and South America, causing an acute-respiratory-distress syndrome (SARS) named COVID-19 by the World Health Organization (WHO). On 12 March 2020, COVID-19 was declared a global pandemic.

In fact, coronaviruses have already been identified in several hosts such as bats, mice, cats, dogs, bulbuls, camels, and other mammals. Most of the coronaviruses that affect humans are associated with fairly mild respiratory symptoms (Drosten et al., 2003) but SARS-CoV-2 appears to be more aggressive and contagious than those previously encountered. Symptoms of SARS-CoV-2 infection include fever, sputum production, headache, fatigue, and shortness of breath, and some patients may report gastrointestinal problems and/or anosmia. The presentation of COVID-19 is very variable, from asymptomatic cases to mild flu-like symptoms, to severe respiratory illness (respiratory failure, septic shock, and multiorgan dysfunction) and death (Huang et al., 2020). Risk factors for severe illness include age, medical comorbidities such as diabetes, cardiovascular diseases, respiratory diseases, and cancer. The very high contagiousness of COVID-19 depends on its rapid community transmission, high virulence, and sustained surface viability.

Coronaviruses are a family of large, single-stranded, enveloped RNA viruses. The RNA-viral genome is contained in a nucleocapsid, which itself is located within a viral envelope (Ortega et al., 2020). The envelope is formed by different proteins such as envelope proteins and membrane proteins. When analyzed using electron microscopy, the virus shows spike proteins that produce a recognizable “crown-like” appearance. These spike proteins are crucial to initiating human infection; the proteins are composed of two unique subunits that allow viral host binding. The S1 domain is involved in the attachment to the host cell membranes, whereas the S2 domain of the spike proteins is responsible for the fusion of the viral and cell host membranes (Li, 2016).

## Angiotensin-Converting Enzyme-2 (ACE2) and Transmembrane Serine Protease 2 (TMPRSS2) Receptors in Human Testes

To enter human cells, SARS-CoV-2 uses angiotensin-converting enzyme-2 (ACE2) as a key receptor. ACE2 is located in different body organs such as kidneys, heart, intestines, liver, lungs, and testes; therefore, cells which have a high expression of ACE2 could be a potential target of the virus. ACE2 is a transmembranal zinc metallopeptidase with a high homology to the classic ACE. ACE isoforms are part of the renin–angiotensin–aldosterone system (RAAS) that plays a crucial role in the regulation of blood pressure and fluid balance. ACE converts Angiotensin I into Angiotensin II (Ang II), whereas ACE2 converts Angiotensin II into Angiotensin 1-7. While Ang II could have dangerous effects on the kidneys, heart, and lungs, the Angiotensin 1-7-Mas receptor axis plays a healthy role because it has vasodilatory, anti-inflammatory, and anti-fibrotic actions (Younis et al., 2020); so even when ACE2 antagonizes the activation of the classical renin–angiotensin system modifying angiotensin II, it still provides some protection against inflammation and fibrosis.

**FIGURE 1:**
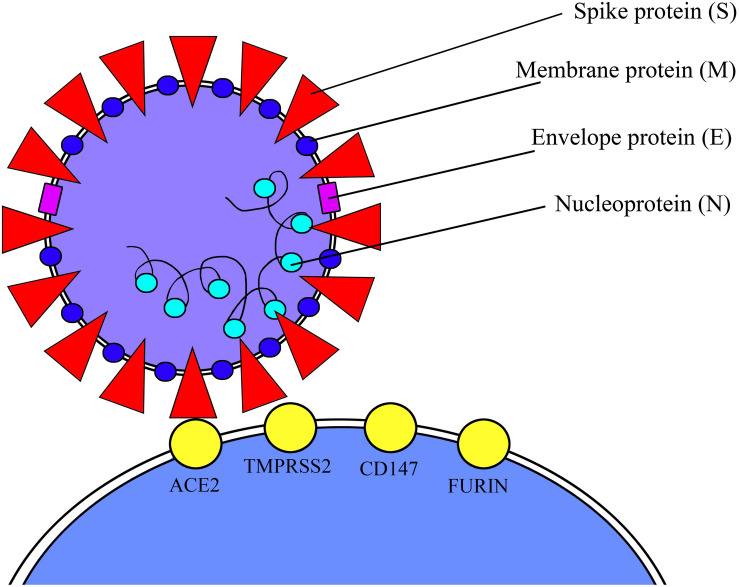
ACE2 and TMPRSS2 viral receptors.

The extracellular domain of ACE2 is a cell surface receptor for the Spikes glycoproteins (S domain) on the SARS-CoV-2 envelope. In order to enter host cells, SARS-CoV-2 uses ACE2 and host proteases such as a transmembrane serine protease 2 (TMPRSS2) that cleaves and induces a conformational change to the viral S domain, allowing the fusion of the viral and host membranes (Hoffmann et al., 2020). In the light of these considerations, both ACE2 and TMPRSS2 have a crucial role for virus host entering (Figure 1). TMPRSS2 is more largely expressed in human tissue than ACE2, whereas single-cell RNA sequencing (scRNAseq) in human respiratory tissue has shown a co-expression of ACE2 and TMPRSS2 in lungs, heart, and kidneys, which would indicate that these cells are strongly susceptible to viral infection (Ding et al., 2004). Given that clinical features of COVID-19 appear to be widely determined by the cells and tissues with co-expression of ACE2 and TMPRSS2 in their constituent cells, it is fitting to evaluate the activity of the virus on those male and female reproductive cells in which there is a co-expression of the two proteins, and if SARS-CoV-2 could consequently have a negative impact on fertility. The expression of ACE2 and TMPRSS2 has been shown in testicular cells with different interpretations (Qi et al., 2020).

In fact, as ACE2 is highly expressed in human testes, it is relevant to evaluate whether COVID-19 in males, via ACE2, might damage fertility. ACE2 is expressed in the testes and epididymis, in particular in Leydig cells, Sertoli cells, and spermatogonia (Wang and Xu, 2020). Moreover, the testicular expression of ACE2 is age-related, with a higher expression in patients aged 20–30, whereas as 60+-year-old patients show a reduced expression of ACE2, it could mean that young men are at higher risk of testicular damage by the virus than older patients. Although ACE2 is the key receptor for the virus, this is not enough to guarantee its entry into the host cell; the co-presence of TMPRSS2 is crucial: *KE.* Stanley et al. (2020) carried out an interesting study based on the evaluation of co-expression of the host virus entry proteins on male reproductive cells.

According to this study, ACE2 is expressed in male myoid cells, spermatogonial stem cells, and Leydig cells, while TMPRSS2 expression is detected predominantly in elongated spermatides. Despite the fact that a small proportion of spermatogonial stem cells express ACE2 and TMPRSS2, finding cells which co-express both genes is extremely rare, and scRNAseq data results suggest that spermatozoa may not be susceptible to virus infection owing to the lack of ACE2 and TMPRSS2 co-expression. Nevertheless, we have to consider that the virus could be present in the seminal fluid, since this not only contains spermatozoa but also round cells (germ stem cells, leukocytes) co-expressing ACE2 and TMPRSS2. Furthermore, since semen is also formed by prostatic components, it would be appropriate to better determine which prostatic cells co-express ACE2 and TMPRSS2. For instance, it is claimed that TMPRSS2 is widely expressed in the prostate epithelial cells, including the apical plasma membrane of the prostate luminal cells and it is also released into seminal fluid as a component of prostasomes, organelle-like vesicles (Chen et al., 2010).

In the light of the evidence, it could be legitimate to assume that the virus via prostatic deriving components and cells could be conveyed into the seminal fluid; however, it has been demonstrated that ACE2 is not expressed on prostatic cells. Table 1 obtained from https://www.proteinatlas.org/humanproteome/sars-cov-2 shows in which male reproductive organs SARS-CoV-2 related proteins are simultaneously expressed, and since, in seminal vesicles, these proteins are simultaneously expressed, virus could be present in the seminal fluid, and semen could be one of the pathways of viral transmission (Gordon et al., 2020; The Human Proteome Atles).

**TABLE 1:** The distribution of SARS-CoV-2 related proteins among male reproductive tissues thus clarifying which cells and reproductive tissues are potential targets of the virus.

***SARS-CoV-2 entry receptors and associated proteases***	**Male reproductive tissue: TESTIS**	**Male reproductive tissue: EPIDIDYMUS**	**Male reproductive tissue: SEMINAL VESICLES**	**Male reproductive Tissue: PROSTATE**
***ACE2***	CELLS IN SEMINIFEROUS DUCTS High expression LEYDIG CELLS Medium expression	NOT DETECTED	GLANDULAR CELLS Low expression	NOT DETECTED
***TMPRSS2***	NOT DETECTED	GLANDULAR CELLS Medium expression	GLANDULAR CELLS Low expression	GLANDULAR CELLS Medium expression
***CTSB*** Is a thiol protease, it is believed to participate in intracellular degradation and turnover of proteins	CELLS IN SEMINIFEROUS DUCTS Low expression LEYDIG CELLS Medium expression	GLANDULAR CELLS High expression	GLANDULAR CELLS High expression	GLANDULAR CELLS High expression
***CTSL*** Is a lysosomal cysteine proteinase that plays a major role in intracellular protein catabolism	NOT DETECTED	GLANDULAR CELLS Low expression	GLANDULAR CELLS Low expression	GLANDULAR CELLS Low expression

*In the seminal vesicles, SARS-CoV-2-related proteins are in glandular cells; this fact suggests that the virus may be internalized in the seminal vesicle cells. Therefore, it is reasonable to hypothesize that the virus could be present in the seminal fluid and it could be sexually transmitted. Data source: https://www.proteinatlas.org/humanproteome/sars-cov-2.*

## SARS-CoV-2 and Repercussions for Male Fertility

SARS-CoV-2 might also indirectly compromise male fertility due to the fever produced and the infiltration of inflammatory molecules that damage Leydig cell functions.

**FIGURE 2:**
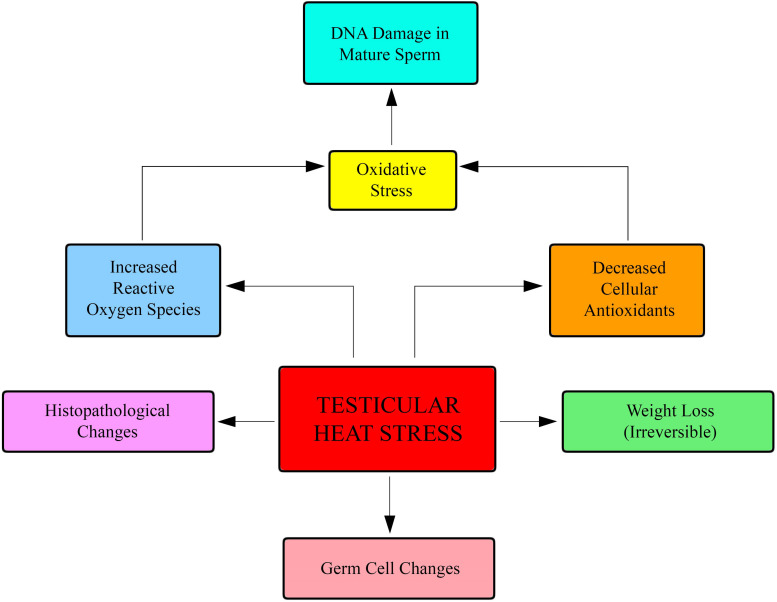
Fever negatively affects fertility. Testicular heat stress causes an increasing of reactive oxygen species that damage sperm DNA.

Apart from finding co-expression of ACE-2 and TMPRSS2 in sperm cells, there would seem to be evidence that COVID-19 could indirectly affect male fertility (Figure 2). One of the major symptoms of the COVID-19 pandemic is a very high fever with a sudden surge in body temperature; fever can negatively affect male fertility. Testicular heat stress determines an increasing of reactive oxygen species, causing oxidative stress and Sperm DNA fragmentation (Albani et al., 2019).

There is additional emerging evidence which would indicate that certain COVID-19 patients might be subject to a secondary cytokine storm syndrome (Loveland et al., 2017; Mehta et al., 2020). This is a hyper-inflammatory syndrome characterized by sustained fever leading to multi-organ failure caused by fatal hyper-cytokinemia. If, on the one hand, cytokines act in order to maintain male reproductive health and testicular function, on the other, COVID-19 determines a change in the cytokine profile and this fact may have further implications for male fertility. Furthermore, immunomodulatory therapies may cause potential long-term effects on male fertility. In addition, cytokine microenvironment deviations within the testes may give rise to adverse tumorigenic effects on the testes (Loveland et al., 2017).

ACE2 is expressed in the testicles, in the Leydig and Sertoli cells. The principal role in the Leydig cells is related to the production of testosterone and sex steroid hormones. Angiotensin 1-7 (obtained thanks to ACE2) could modulate testosterone secretion (Leal et al., 2009; Reis et al., 2010).

Although the testicular expression of ACE2 could point to the opinion that the virus might possibly enter into the testicles, not all literature is concordant.

In fact, Song et al. (2020b) collected 12 semen samples from surviving COVID-19 patients and testicular biopsies from one deceased COVID-19 patient, and it was found that SARS-CoV-2 RNA was detected neither in the semen samples nor in the testicular tissue biopsy. Nevertheless, it might be possible to find a condition of infertility in patients who have acquired the SARS-CoV-2 infection, because this could cause an indirect inflammatory/immune response in the testicles.

Xu et al. (2006) observed a high concentration of inflammatory molecules in the testicles of patients infected by SARS-CoV. The inflammatory cells and molecules could interfere with the activity of the Leydig cells, thereby impeding testosterone production, as well as destroying seminiferous tubule cells. The cytokines produced by inflammatory cells could provoke an auto-immune response within the seminiferous tubules. However, in an acute phase, these alterations may not be evident.

Ma et al. (2020), in a retrospective study on COVID-19 patients, stressed the fact that patients showed a notably higher serum luteinizing hormone (LH) and prolactin than healthy men, but no changes in the testosterone serum levels were detected. Impaired testosterone production in the early stage might probably determine LH release, which could temporarily maintain the testosterone level, thus clinical hypogonadism and consequent infertility might emerge only later.

## Taking the Above Considerations Into Account, Is SARS-CoV-2 Viable in Semen? Could Seminal Fluid Represent a Path for the Virus to Be Sexually Transmitted?

Considering the COVID-19 global pandemic, it is imperative to identify all possible routes of transmission of the virus. Even though there is skepticism about the transmission of SARS-CoV-2 via seminal fluid, all possibilities have to be considered; after all, over 25 viruses have been detected in human semen, and sexual transmission has been confirmed in several viruses traditionally considered to be non-sexually transmitted. Viruses able to cause viremia can pass the blood-testes barriers (e.g., anti-sperm antibodies) and can survive in the male reproductive system because the testicular immune response is related to sperm survival (Li et al., 2012; Salam and Horby, 2017). Moreover, human viral infections such as sexually transmitted infection, tuberculosis, and mumps are known to be able to damage the testes. In fact, a recent study of COVID-19 positive men demonstrated that 19% of participants experienced scrotal discomfort similar to that suffered by males with orchitis (Pan et al., 2020). Nevertheless, the presence of SARS-CoV-2 in semen and sperm needs to be better evaluated. The few studies about the presence of the virus in the semen have shown controversial results so far. Two studies concerning COVID-19-positive patients were unable to detect the presence of the virus in semen (Pan et al., 2020; Song et al., 2020a). However, a study of 38 male patients with COVID-19 found that four of 15 patients in the acute stage of infection, and two of 23 patients in recovery, had detectable SARS-CoV-2 present in their semen samples. There is no literature about the presence of the virus in the semen of asymptomatic men; therefore, it would be advantageous to evaluate the presence of the virus in the semen samples of males with varying degrees of illness, both with and without symptoms. Since there is no evidence about SARS-CoV-2 sexual transmission, it would be reasonable to better investigate not only about the presence of the virus in semen but also in vaginal fluid. Qiu et al. (2020) carried out a study finalized to detect the presence of SARS-CoV-2 in the vaginal fluid of 10 COVID-19 positive women; as a result, no SARS-CoV-2 virus was noted. Nevertheless, only postmenopausal women were evaluated and the vaginal swabs were analyzed 17 days or more after disease onset; this fact makes it difficult to assess the presence of the virus by reverse transcription-polymerase chain reaction (RT-PCR). Lower viral spreading to genital organs and genital secretions may be due to low rate of viremia for COVID-19. Besides, an analysis concerning the expression of SARS-CoV-2-related proteins reveals that they are not present in the female reproductive organs, and this suggests that the female reproductive organs are not viral targets as shown in Table 2 obtained from https://www.proteinatlas.org/humanproteome/sars-cov-2 (Gordon et al., 2020; The Human Proteome Atles).

**TABLE 2:** The distribution of SARS-CoV-2-related proteins among female reproductive tissues, thus clarifying which cells and reproductive tissues are potential targets of the virus.

***SARS-CoV-2 entry receptors and associated proteases***	**Female reproductive system: OVARY**	**Female reproductive system: VAGINA**	**Female reproductive tissue: ENDOMETRIUM**	**Female reproductive system: FALLOPIAN TUBE**
*ACE2*	NOT DETECTED	NOT DETECTED	NOT DETECTED	NOT DETECTED
*TMPRSS2*	NOT DETECTED	NOT DETECTED	NOT DETECTED	NOT DETECTED
*CTSB* Is a thiol protease, it is believed to participate in intracellular degradation and turnover of proteins	FOLLICLE CELLS High expression	SQUAMOUS EPITHELIAL CELLS Low expression	ENDOMETRIAL STROMA Medium expression	GLOBULAR CELLS High expression
*CTSL* Is a lysosomal cysteine proteinase that plays a major role in intracellular protein catabolism	NOT DETECTED	NOT DETECTED	NOT DETECTED	NOT DETECTED

*It is remarkable that ACE2 and TMPRSS2 are not expressed in the ovary, fallopian tubes, endometrium, and vagina. This evidence suggests that these organs are not viral targets. Data source: https://www.proteinatlas.org/humanproteome/sars-cov-2.*

However, the risk of a viral contamination due to genital fluids, even with low rate, is not unexpected. Delfino et al. (2020) have suggested to perform routinely RT-PCR assays for SARS-CoV-2 detection on three swabs, nasopharyngeal, vaginal, and rectal, aiming to reduce the risk of sexual transmission. In fact, it has been demonstrated that despite a negative nasopharyngeal testing, COVID-19 patients can persistently result positive on rectal swabs (Yuksel and Ozgor, 2020). In the light of aforementioned considerations and in view of the fact that intestinal cells express SARS-CoV-2 viral receptors, the SARS-CoV-2 transmission, as well as HIV transmission, could occur via sexual anogenital and orogenital contacts (Semprini et al., 1992).

## Gender Differences in Patients With COVID-19: Why Do Men Appear More Susceptible Than Women?

Recently, many different studies have demonstrated that men suffer worse clinical outcomes and COVID deaths than women. A variety of factors may cause disparities in sex-specific disease outcomes. For instance, sex-specific differences could be related to steroids and X-linked genes; it is well known that men and women differ in both innate and adaptive immune responses (Jin et al., 2020). Sex-specific inflammatory responses provoked by the unique mode of inheritance of the X chromosome may lead to the gender differences (Meng et al., 2020). The immune genes located on the X chromosome would help to generate a lower inflammatory response, while CD4 + levels are higher in women with an enhancement of the immune response. Moreover, women produce higher levels of antibodies which remain in circulation for a longer period of time, and besides, activation levels of the immune cells are higher. This fact is related to Toll-like receptor 7 (TLR7) and to the production of interferon gamma (INFγ). TLR7 is higher and biallelic in women, which means that the female immune response is more enhanced than in men.

TLR7 is expressed by innate immunity cells; it recognizes viral RNA, promoting the production of antibodies and inflammatory cytokines such as IL6. Nevertheless, in women, the production of IL6 is lower than in men, and it is linked to greater female longevity (Younis et al., 2020).

Genetic and hormonal differences may give rise to higher male susceptibility. Although the etiology of COVID-19 is probably multifactorial (work/environment factors, gender defense factors), one of the reasons for this different gender vulnerability could also be related to the production of androgens. According to La Vignera et al., androgens sustain the expression of ACE2; this fact may explain the reasons why men are more exposed to the virus. In fact, prostate cancer patients undergoing Androgen Deprivation Therapy (ADT) show a lower expression of ACE2; therefore, ADT could deter SARS-CoV-2 penetration (La Vignera et al., 2020). Eventually, available literature suggests that androgens lead to the generation of an overexpression of TMPRSS2 too, which is crucial for the virus to be able to infect (Lamy et al., 2020; La Vignera et al., 2020).

## Conclusion

The pandemic due to SARS-CoV-2 has generated increasing concerns about male fertility and reproduction. The evidences in the literature are very controversial, and further studies are needed to evaluate if the virus can damage male reproductive capability. It is argued that testicular cells expressing both ACE2 and TMPRRS2 are rare, and this fact suggests that the virus may not harm male gametes. However, SARS-CoV-2 could indirectly compromise male gametes, testicular cells, and therefore fertility because the fever and the cytokine storm associated with COVID-19 induce a sperm DNA fragmentation and reduce the male reproductive potential. Finally, although the evidence is controversial, semen could be a viral transmission way. The seminal fluid is formed by several components that not only derive from the testicles but also from the prostate; in fact, it has been suggested that some prostatic cells express TMPRSS2 and release it onto the seminal fluid. This evidence leads to speculation about the fact that semen could represent a SARS-CoV-2 transmission way. The Italian Society of Andrology and Sexual Medicine (SIAMS) stresses the need to carry out further studies aimed at uniquely determining the effects of the virus on testicular functions and detecting its presence in the seminal fluid (Corona et al., 2020). In the COVID-19 era, conclusive evidence about SARS-CoV-2 transmission by spermatozoa has not been established. Therefore, in order to perform assisted reproductive procedures, it would be appropriate to preventively treat the semen sample processing it, according to the procedure used for HIV-positive semen samples (Semprini et al., 1992).

The evaluation of the presence of SARS-CoV-2 in the seminal fluid is also very important for the semen cryopreservation procedures in liquid nitrogen since viruses stored in liquid nitrogen retain their pathogenic properties (De Paoli, 2005). Finally, with the aim of understanding if the virus could be sexually transmitted, it could be reasonable to establish whether vaginal fluid represents a transmission path too, and since SARS-CoV-2 shows analogies with the HIV virus, it is legitimate to evaluate whether sexual oral-anal contacts could significantly increase the risk of COVID-19 transmission. Further investigation will be crucial in order to better know a virus that is causing devastating effects on the worldwide population.

## Author Contributions

AN designed the review, carried out an analysis of scientific literature about SARS-CoV-2 and male fertility, and wrote the manuscript. EA evaluated cellular lines in testes and seminal fluid. SC and LA carried out a study concerning spermatozoa and oxidative stress. PL-S analyzed the data and manuscript. All authors contributed to the article and approved the submitted version.

## Conflict of Interest

The authors declare that the research was conducted in the absence of any commercial or financial relationships that could be construed as a potential conflict of interest.

## References

[B1] Albani E. Castellano S. Gurrieri B. Arruzzolo L. Negri L. Borroni E. M. (2019). Male age: negative impact on sperm DNA fragmentation. *Aging* 11 2749–2761. 10.18632/aging.101946 31085803 PMC6535069

[B2] Chen Y. W. Lee M. S. Lucht A. Chou F. P. Huang W. Havighurst T. C. (2010). TMPRSS2, a serine protease expressed in the prostate on the apical surface of luminal epithelial cells and released into semen in prostasomes, is misregulated in prostate cancer cells. *Am. J. Pathol.* 176 2986–2996. 10.2353/ajpath.2010.090665 20382709 PMC2877858

[B3] Corona G. Baldi E. Isidori A. M. Paoli D. Pallotti F. De Santis L. (2020). SARS-CoV-2 infection, male fertility and sperm cryopreservation: a position statement of the Italian Society of Andrology and Sexual Medicine (SIAMS) (Società Italiana di Andrologia e Medicina della Sessualità). *J. Endocrinol. Invest.* 10.1007/s40618-020-01290-w [Epub ahead of print]. PMC7252417 32462316

[B4] De Paoli P. (2005). Bio-banking in microbiology: from sample collection to epidemiology, diagnosis and research. *FEMS Microbiol. Rev.* 29 897–910. 10.1016/j.femsre.2005.01.005 16219511 PMC7110355

[B5] Delfino M. Guida M. Patrì A. Spirito L. Gallo L. Fabbrocini G. (2020). SARS-CoV-2 possible contamination of genital area: implications for sexual and vertical transmission routes. *J. Eur. Acad. Dermatol. Venereol.* 34 e364–e365. 10.1111/jdv.16591 32379909 PMC7267668

[B6] Ding Y. Li H. Zhang Q. Huang Z. Che X. Hou J. (2004). Organ distribution of severe acute respiratory syndrome (SARS) associated coronavirus (SARS-CoV) in SARS patients: implications for pathogenesis and virus transmission pathway. *J. Pathol.* 203 622–630. 10.1002/path.1560 15141376 PMC7167761

[B7] Drosten C. Günther S. Preiser W. van der Werf S. Brodt H. R. Becker S. (2003). Identification of a novel coronavirus in patients with severe acute respiratory syndrome. *N. Engl. J. Med.* 348 1967–1976. 10.1056/NEJMoa030747 12690091

[B8] Gordon D. E. Jang G. M. Bouhaddou M. Xu J. Obernier K. White K. M. (2020). A SARS-CoV-2 protein interaction map reveals targets for drug repurposing. *Nature* 583 459–468. 10.1038/s41586-020-2286-9 32353859 PMC7431030

[B9] Hoffmann M. Kleine-Weber H. Schroeder S. Krüger N. Herrler T. Erichsen S. (2020). SARS-CoV-2 cell entry depends on ACE2 and TMPRSS2 and is blocked by a clinically proven preotease inhibitor. *Cell* 181 271.e8–280.e8. 32142651 10.1016/j.cell.2020.02.052 PMC7102627

[B10] Huang C. Wang Y. Li X. Ren L. Zhao J. Hu Y. (2020). Clinical features of patients infected with 2019 novel coronavirus in Wuhan, China. *Lancet* 395 497–506. 10.1016/S0140-6736(20)30183-5 31986264 PMC7159299

[B11] Jin J. M. Bai P. He W. Wu F. Liu X. F. Han D. M. (2020). Gender differences in patients with COVID-19: focus on severity and mortality. *Front. Public Health* 8:152. 10.3389/fpubh.2020.00152 32411652 PMC7201103

[B12] La Vignera S. Cannarella R. Condorelli R. A. Torre F. Aversa A. Calogero A. E. (2020). SARS-CoV-2: the endocrinological protective clinical model derived from patients with prostate cancer. *Ther. Adv. Endocrinol. Metab.* 11:2042018820942385. 10.1177/2042018820942385 PMC7357014 32699587

[B13] Lamy P. J. Rébillard X. Vacherot F. de la Taille A. (2020). Androgenic hormones and the excess male mortality observed in COVID-19 patients: new convergent data. *World J. Urol.* 10.1007/s00345-020-03284-y [Epub ahead of print]. 32488360 PMC7266423

[B14] Leal M. C. Pinheiro S. V. Ferreira A. J. Santos R. A. Bordoni L. S. (2009). The role of angiotensin- (1-7) receptor Mas in spermatogenesis in mice and rats. *J. Anat.* 214 736–743. 10.1111/j.1469-7580.2009.01058.x 19438767 PMC2707096

[B15] Li F. (2016). Structure, function and evolution of coronavirus spike proteins. *Annu. Rev. Virol.* 3 237–261. 10.1146/annurev-virology-110615-042301 27578435 PMC5457962

[B16] Li N. Wang T. Han D. (2012). Structural, cellular and molecular aspects of immune privilege in the testis. *Front. Immunol.* 3:152. 10.3389/fimmu.2012.00152 22701457 PMC3371599

[B17] Loveland K. L. Klein B. Pueschl D. Indumathy S. Bergmann M. Loveland B. E. (2017). Cytokines in male fertility and reproductive pathologies: immunoregulation and beyond. *Front. Endocrinol.* 8:307. 10.3389/fendo.2017.00307 29250030 PMC5715375

[B18] Ma L. Xie W. Li D. Shi L. Mao Y. Xiong Y. (2020). Effects of SARS_CoV-2 infection upon male gonadal function; a sible center based study. *medRxiv* [Preprint]. 10.1101/2020.03.21.20037267

[B19] Mehta P. McAuley D. F. Brown M. Sanchez E. Tattersall R. S. Manson J. J. (2020). COVID-!9: consider cytokine storm syndromes and immunosuppression. *Lancet* 395 1033–1034. 10.1016/s0140-6736(20)30628-0 32192578 PMC7270045

[B20] Meng Y. Wu P. Lu W. Liu K. Ma K. Huang L. (2020). Sex-specific clinical characteristics and prognosis of coronavirus disease-19 infection in Wuhan, China: a retrospective study of 168 severe patiens. *PLoS Pathog.* 16:e1008520. 10.1371/journal.ppat.1008520 32343745 PMC7209966

[B21] Ortega J. T. Serrano M. L. Pujol F. H. Rangel H. R. (2020). Role of chenges in SARS-CoV-2 spike protein in the interaction with hte human ACE2 receptor: an in silico analysis. *EXCLI J.* 19 410–417. 32210742 10.17179/excli2020-1167 PMC7081066

[B22] Pan F. Xiao X. Guo J. Song Y. Li H. Patel D. P. (2020). No evidence of SARS-CoV-2 in semen of males recovering from COVID-19. *Fertil. Steril.* [Epub ahead of print]. 10.1016/j.fertnstert.2020.04.024 PMC7164916 32482249

[B23] Qi J. Zhou Y. Hua J. Zhang L. Bian J. Liu B. (2020). The scRNA-seq expression profiling of the receptor ACE2 and the cellular protease TMPRSS2 reveals human organs susceptible to COVID-19 infection. *bioRxiv* [Preprint]. 10.1101/2020.04.16.045690 PMC7794913 33401657

[B24] Qiu L. Liu X. Xiao M. Xie J. Cao W. Liu Z. (2020). SARS-CoV-2 is not detectable in the vaginal fluid of women with severe COVID-19 infection. *Clin. Infect. Dis.* 71 813–817. 10.1093/cid/ciaa375 32241022 PMC7184332

[B25] Reis A. B. Araújo F. C. Pereira V. M. Dos Reis A. M. Santos R. A. Reis F. M. (2010). Angiotensin(1-7) and its receptor Mas are expressed in the human testis: implication for male inferitlity. *J. Mol. Histol.* 41 75–80. 10.1007/s10735-010-9264-8 20361351

[B26] Salam A. P. Horby P. W. (2017). The breadth of viruses in human semen. *Emerg. Infect. Dis.* 23 1922–1924. 10.3201/eid2311.171049 29048276 PMC5652425

[B27] Semprini A. E. Levi-Setti P. Bozzo M. Ravizza M. Taglioretti A. Sulpizio P. (1992). Insemination of HIV-negative women with processed semen of HIV-positive partners. *Lancet* 340 1317–1319. 10.1016/0140-6736(92)92495-2 1360037

[B28] Song C. Wang Y. Li W. Hu B. Chen G. Xia P. (2020a). Absence of 2019 novel coronavirus in semen and testes of COVID-19 patients. *Biol. Reprod.* 16:ioaa050. 10.1093/biolre/ioaa050 PMC7184456 32297920

[B29] Song C. Wang Y. Li W. Hu B. Chen G. Xia P. (2020b). Detection of 2019 novel coronavirus in semen and testicular bipsy specimen of COVID-19 patients. *medRxiv* [Preprint]. 10.1101/2020.03.31.20042333

[B30] Stanley K. E. Thomas E. Meaver M. Wells D. (2020). Corovirus disease (COVID-19) and fertility: viral host entry protein expression in male and female reproductive tissues. *Fertil. Steril.* 114 33–43. 10.1016/j.fertnstert.2020.05.001 32622411 PMC7205710

[B31] The Human Proteome Atles (0000). *The Human Protein Atlas.* Available online at: https://www.proteinatlas.org/humanproteome/sars-cov-2

[B32] Wang Z. Xu X. (2020). ScRNA-seq profiling of human testes reveals the presence of the ACE2 receptor, a target for SARS-CoV-2 Infection in spermatogonia, leydig and sertoli cells. *Cells* 9:920. 10.3390/cells9040920 32283711 PMC7226809

[B33] Wu Z. McGoogan J. M. (2020). Characteristics of and Important Lessons from the Corovirus disease 2019 (COVID-19) outbreak in China: summary of a Report of 72 314 Cases from the Chinese Center for Disease Control and Prevention. *JAMA* 323, 1239–1242. 10.1001/jama.2020.2648 32091533

[B34] Xu J. Q. L. Chi X. Yang J. Wei X. Gong E. Peh S. (2006). Orchitis: a complication of severe acute respiratory syndrome (SARS). *Biol. Reprod.* 74 410–416. 10.1095/biolreprod.105.044776 16237152 PMC7109827

[B35] Younis J. S. Abassi Z. Skorecki K. (2020). Is there an impact of the COVID-19 pandemic on male fertility? The ACE 2connection. *Am. J. Physiol. Endocrinol. Metab.* 318 E878–E880. 10.1152/ajpendo.00183.2020 32421367 PMC7276979

[B36] Yuksel B. Ozgor F. (2020). Effect of the COVID-19 pandemic on female sexual behavior. *Int. J. Gynaecol. Obstet.* 150 98–102. 10.1002/ijgo.13193 32392400 PMC9087619

